# A precise and efficient circular RNA synthesis system based on a ribozyme derived from *Tetrahymena thermophila*

**DOI:** 10.1093/nar/gkad554

**Published:** 2023-06-28

**Authors:** Jingyi Cui, Lanxin Zhang, Zaifeng Zhang, Xuanmei Luo, Ye Liu, Chang Li, Wei Huang, Lihui Zou, Xue Yu, Fei Xiao

**Affiliations:** The Key Laboratory of Geriatrics, Beijing Institute of Geriatrics, Institute of Geriatric Medicine, Chinese Academy of Medical Sciences, Beijing Hospital/National Center of Gerontology of National Health Commission, PR China; Graduate School of Peking Union Medical College, Beijing 100730, PR China; Clinical Biobank, Beijing Hospital, National Center of Gerontology, Institute of Geriatric Medicine, Chinese Academy of Medical Sciences, PR China; Clinical Biobank, Beijing Hospital, National Center of Gerontology, Institute of Geriatric Medicine, Chinese Academy of Medical Sciences, PR China; The Key Laboratory of Geriatrics, Beijing Institute of Geriatrics, Institute of Geriatric Medicine, Chinese Academy of Medical Sciences, Beijing Hospital/National Center of Gerontology of National Health Commission, PR China; Graduate School of Peking Union Medical College, Beijing 100730, PR China; The Key Laboratory of Geriatrics, Beijing Institute of Geriatrics, Institute of Geriatric Medicine, Chinese Academy of Medical Sciences, Beijing Hospital/National Center of Gerontology of National Health Commission, PR China; The Key Laboratory of Geriatrics, Beijing Institute of Geriatrics, Institute of Geriatric Medicine, Chinese Academy of Medical Sciences, Beijing Hospital/National Center of Gerontology of National Health Commission, PR China; The Key Laboratory of Geriatrics, Beijing Institute of Geriatrics, Institute of Geriatric Medicine, Chinese Academy of Medical Sciences, Beijing Hospital/National Center of Gerontology of National Health Commission, PR China; The Key Laboratory of Geriatrics, Beijing Institute of Geriatrics, Institute of Geriatric Medicine, Chinese Academy of Medical Sciences, Beijing Hospital/National Center of Gerontology of National Health Commission, PR China; The Key Laboratory of Geriatrics, Beijing Institute of Geriatrics, Institute of Geriatric Medicine, Chinese Academy of Medical Sciences, Beijing Hospital/National Center of Gerontology of National Health Commission, PR China; Department of Cardiology, Beijing Hospital, National Center of Gerontology, Institute of Geriatric Medicine, Chinese Academy of Medical Sciences, PR China; The Key Laboratory of Geriatrics, Beijing Institute of Geriatrics, Institute of Geriatric Medicine, Chinese Academy of Medical Sciences, Beijing Hospital/National Center of Gerontology of National Health Commission, PR China; Graduate School of Peking Union Medical College, Beijing 100730, PR China; Clinical Biobank, Beijing Hospital, National Center of Gerontology, Institute of Geriatric Medicine, Chinese Academy of Medical Sciences, PR China

## Abstract

Classic strategies for circular RNA (circRNA) preparation always introduce large numbers of linear transcripts or extra nucleotides to the circularized product. In this study, we aimed to develop an efficient system for circRNA preparation based on a self-splicing ribozyme derived from an optimized *Tetrahymena thermophila* group Ⅰ intron. The target RNA sequence was inserted downstream of the ribozyme and a complementary antisense region was added upstream of the ribozyme to assist cyclization. Then, we compared the circularization efficiency of ribozyme or flanking intronic complementary sequence (ICS)-mediated methods through the *DNMT1*, *CDR1as*, *FOXO3*, and *HIPK3* genes and found that the efficiency of our system was remarkably higher than that of flanking ICS-mediated method. Consequently, the circularized products mediated by ribozyme are not introduced with additional nucleotides. Meanwhile, the overexpressed circFOXO3 maintained its biological functions in regulating cell proliferation, migration, and apoptosis. Finally, a ribozyme-based circular mRNA expression system was demonstrated with a split green fluorescent protein (GFP) using an optimized Coxsackievirus B3 (CVB3) internal ribosome entry site (IRES) sequence, and this system achieved successful translation of circularized mRNA. Therefore, this novel, convenient, and rapid engineering RNA circularization system can be applied for the functional study and large-scale preparation of circular RNA in the future.

## Graphical Abstract

**Graphical Abstract:**
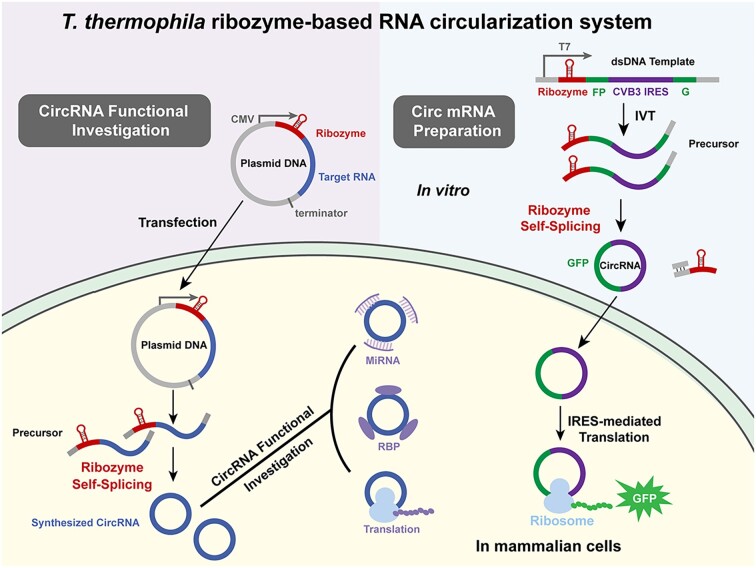

## INTRODUCTION

Circular RNAs (circRNAs) are a class of covalently closed RNA molecules produced from precursor mRNA back splicing and are widely found in nature (1). Due to the lack of 5' or 3' ends, circRNAs have a certain resistance to exonuclease digestion, which makes circRNAs more stable than linear RNAs. Most natural circRNAs are noncoding, although a few of them can be translated into peptides (2,3). Noncoding circRNAs can serve as sponges and influence corresponding functions by binding to miRNAs and proteins (4). Endogenous circRNAs are essential in normal cell differentiation, tissue homeostasis, and disease development (5). In addition, some endogenous circRNAs play a role in antiviral responses while some are associated with immune responses. Exogenous circRNAs can stimulate immune signaling in mammalian cells by activating the pattern-recognition receptor RIG-I (6).

CircRNAs can be used to express various types of proteins and perform the same function as mRNAs, which makes them a promising next-generation mRNA therapy (7). CircRNAs can overcome some limitations of mRNA in terms of stability, nonspecific tissue expression, and immunogenicity (8). Ubiquitous RNA enzymes in the environment threaten mRNA stability due to their open ends (9). In contrast, with their ring structures, circRNAs are resistant to the RNA degradation system *in vivo*, which leads to a longer half-life for their effects as a therapeutic drug. CircRNAs can also be designed and optimized for effective expression in specific tissues and cells (10,11), which helps to reduce the side effects of circRNAs even if they are delivered into improper tissues. Furthermore, mRNAs have immunogenicity that can excessively activate innate immunity, resulting in symptoms similar to those of viral infection, inflammation, and autoimmune diseases (12,13). In contrast, circRNAs display relatively low immunogenicity even without base modification (14,15), showing more advantages in the research and development of future patented drugs and vaccines.

Currently, there are two main strategies for engineering RNA circularization *in vitro*: T4 RNA/DNA ligase ligation or ribozyme methods. The ligases used for circularization can be either T4 DNA ligase or T4 RNA ligases 1 and 2. All three enzymes are ATP-dependent and can catalyze the binding of 5'-phosphate and 3'-hydroxyl groups at the end of the RNA. However, ligase-mediated circularization is not suitable for the construction of long fragments due to its low circularization efficiency, abundant byproducts with exogenous nucleotides, and complicated preparation and purification processes (16). Previously, some catalytic RNAs have been widely applied to synthesize larger circRNAs. A group of small RNAs derived from satTRSV (-) RNA was designed to produce self-circularizing RNAs of known sizes (17). The hairpin ribozyme variants have also been engineered to generate circular RNA (18). Another common method called permuted intron-exon (PIE) relies on a spontaneous group I intron self-splicing system derived from the thymidylate synthase gene of the T4 phage, pretRNA_Leu_ of *Anabaena* or pre-rRNA of *Tetrahymena* (19–22). The permuted group I intron precursor RNA contains end-to-end fused exons that interrupt half intron sequences. Foreign sequences can be integrated into the exon of such a permuted self-splicing system, thus allowing the synthesis of circRNAs. Compared with the T4 RNA ligase method, the group I intron ribozyme-based circularization method is more suitable for the synthesis of long fragment circRNA and substantially improves efficiency. However, the latter also has many problems. For example, circRNA derived from the phage T4 thymidylate synthase (td) gene or permuted Anabaena pretRNA group I intron cannot clip off the long splints that assist circular formation, thus introducing an additional 74 nt or 186 nt of nucleotides, respectively (21). The most common method of endogenic RNA circularization for circRNA function research in mammalian cells is mediated by flanking complementary sequences, such as ALU repeats or short intronic complementary sequences. For the flanking ICS-mediated circularization method, endonucleolytic splicing for generating RNA fragments appears to be a rate-limiting step. Due to its low efficiency, the flanking ICS method may lead to the introduction of a large number of linear transcripts, which may exert nonspecific effects in the studies of biological function.

In the present study, we used an optimized ribozyme from the *Tetrahymena therm*ophila group I intron (23–25) to establish a novel and simplified system for RNA circularization both in mammalian cells and *in vitro*, generating an ideal circRNA without any additional nucleotides for the overexpression in cells, and therefore, this system is suitable for large-scale industrial production of circular mRNA.

## MATERIALS AND METHODS

### Vector construction

The active ribozyme contained the sequences from 28 to 414 nt of the *T. thermophila* intron, which was located in the intervening region of the *T. thermophila* large subunit rRNA precursor. The sequences of the *Tetrahymena* ribozyme with an internal guide sequence (IGS), the RNAs to be circularized, and a 45-nt antisense region were chemically synthesized by Beijing Genomics Institute (Beijing, China) and cloned into a double-enzyme-digested linearized pCDH vector.

The overexpression plasmids of specific circRNAs based on flanking ICSs and paired control plasmids were obtained from the Public Protein/Plasmid Library (PPL, Nanjing, China), which contains a general front and back circular frame. The indicated RNA sequences were cloned into the construct by EcoRI/BamHI restriction sites.

For the GFP reporter gene vector, a synthesized Coxsackievirus B3 (CVB3) internal ribosome entry site (IRES) fragment and an exon that encoded GFP were inserted into the abovementioned circRNA overexpression vector using the *Tetrahymena* ribozyme. Specifically, the exon was divided into two parts, and the split GFP gene fragments were inserted into the vector in reverse order. The CVB3 IRES was then inserted between the split GFP gene start and stop codons, resulting in a large exon downstream of the ribozyme. Similarly, we used Gibson Assembly to obtain the full-length sequence. The primers and the sequences of these constructs are listed in Supplementary Table S1.

### Cell culture and transfection

Human cervical cancer (HeLa), breast cancer (MCF7), HEK293T, colon cancer (HCT-8, HCT-116), and prostate cancer (DU145 and PC-3) cell lines were purchased from the American Type Culture Collection (ATCC, VA, USA). HeLa, MCF7, HEK293T and HCT-116 cells were cultured in DMEM containing 10% fetal bovine serum (FBS) (Gibco, MA, USA), while HCT-8, PC-3 and DU145 cells were maintained in RPMI 1640 medium with 10% FBS. All cells were cultured at 37 °C in a humidified incubator containing 5% CO_2_ to a density of 70%-80% and were transfected with Lipo8000 Transfection Reagent (Beyotime Biotechnology, Shanghai, China) or Lipofectamine 3000 reagent (Invitrogen, CA, USA).

### *In vitro* transcription and RNA circularization

DNA templates for *in vitro* transcription were amplified, and an upstream T7 promoter was added by PCR using Phanta Flash Master Mix (Vazyme, Nanjing, China). The product was identified with 1.2% agarose gel electrophoresis and purified with a TIANgel purification kit (Tiangen, Beijing, China). T7 RNA polymerase (NEB, MA, USA) was used for *in vitro* transcription reactions that lasted 2 h at 37°C. After *in vitro* transcription, the RNA products were treated with DNase I (Beyotime Biotechnology, Shanghai, China) at 37°C for 30 min to remove DNA templates. Each circularization reaction was supplemented with 2 mM GTP and incubated at 55°C for 15 min to induce RNA circularization, followed by quick cooling to 4°C. The acetate/ethanol precipitation method was used to purify the RNA (26).

### RNA extraction, reverse transcription, and polymerase chain reaction (PCR)

Total RNA was extracted using TRIzol reagent (Invitrogen, CA, USA) according to the manufacturer's instructions and subsequently treated with DNase I (Beyotime Biotechnology, Shanghai, China) at 37°C for 15 min, followed by treatment at 65°C for 10 min to inactivate DNase I. The OD value 260/280 of the extracted RNA was between 1.9 and 2.1 and the OD value 260/230 was greater than 2.0. One microgram of RNA was reverse-transcribed using Evo M-MLV RT Premix (Accurate Biology, Hunan, China) according to the manufacturer's instructions. The RT product was used for RT-PCR amplification with Phanta Flash Master Mix (Vazyme, Nanjing, China). The RT-PCR product was then separated on a 1.2% agarose gel and scanned with a scanner (Bio-Rad, CA, USA). The same cDNA template was diluted 4-fold for quantitative real-time PCR (qPCR) with a SYBR Green Premix Pro Taq HS qPCR kit (Accurate Biology, Hunan, China). The reaction procedure for qPCR was incubation at 95°C for 30 s, followed by 40 cycles of 95°C for 5 s and 58°C for 30 s on an iQ5 Real-Time PCR Thermal Cycler (Bio-Rad, CA, USA). The 2^−ΔΔCt^ method was used to calculate relative fold changes. The gene expression levels were normalized to the endogenous expression of the housekeeping gene *GAPDH*. The splicing efficiency value of circRNA was calculated by dividing circRNA by total RNA products (circRNA/total RNA). The error bar represents the standard error of the mean (SEM) of three independent experiments. The primers used for the PCR are listed in Supplementary Table S1.

### Northern blotting (NB)

In brief, RNA was resolved on formaldehyde denatured agarose gel, followed by transfer to a nylon membrane. Digoxigenin (Dig)-labelled cDNA probes were made using a DIG DNA Labelling kit (Mylab, Beijing, China). The membrane was then hybridized with specific Dig-labelled probes at 42°C. The NB probe is listed in Supplementary Table S1. Detection steps were performed using a DIG hybrid detection kit (Mylab, Beijing, China) according to the manufacturer's protocol.

### Immunoblotting

The cells were washed twice with phosphate-buffered saline (PBS), and total protein was extracted with a Whole Protein Extraction Kit (Keygen, Nanjing, China). Protein samples were separated by SDS−PAGE and then transferred to PVDF membranes. Subsequently, the membranes were blocked in 1 × casein buffer for 1 h at room temperature and then incubated with anti-cleaved-parp1 antibody (Santa Cruz, CA, USA), anti-cleaved-caspase 3 antibody (Cell Signaling Technology, USA), anti-GAPDH antibody (Abmart, Shanghai, China) and anti-β-actin antibody (Abmart, Shanghai, China) at 4°C overnight. The membranes were washed three times with TBS-T, incubated with secondary antibodies (Abmart, Shanghai, China) for 1 h, and detected with a gel imaging analysis system (Sinsage, Beijing, China).

### Cell viability assay

A total of 3  ×  10^3^ transfected cells were harvested and plated in 100 μl of medium containing 10% FBS per well in 96-well plates. Then, 100 μl of medium containing 10% Cell Counting Kit-8 (CCK-8) reagent (Meilunbio, Dalian, China) was added to each well at different time points (0, 24, 48, 72 or 96 h). Then, the cells were incubated at 37°C for 1 h. The absorbance at 450 nm was measured by a multimode microplate reader (BioTek, Bedfordshire, UK).

### Colony formation assay

A total of 1.0  × 10^3^ transfected cells were seeded in each well of a 6-well plate and cultured at 37°C for 14 days. On the final day in culture, the cells were fixed for 15 min with methanol and then washed twice with PBS. Then, the cells were stained for 30 min with a 0.5% crystal violet solution. The colonies were photographed and counted with ImageJ software. Alternatively, acetic acid was used to wash off the crystal violet, and was taken the eluent to measure the OD value at 570 nm on a multimode microplate reader (BioTek, Bedfordshire, UK).

### Transwell assay

A 24-well transwell insert with an upper chamber was used. Briefly, 1.0 × 10^5^ transfected cells in 200 μl of serum-free medium were seeded in the upper chamber, and 600 μl of medium containing 10% FBS was added to the lower chamber. After incubation at 37°C for 24 h, the cells on the upper membrane surface were removed with a cotton swab, and the cells that had migrated to the bottom surface of the upper membrane were fixed in methanol for 30 min and then stained with 0.5% crystal violet for 30 min. The number of migrating cells was counted with ImageJ software. Alternatively, acetic acid was used to wash off the crystal violet, and the eluent was taken to measure the OD value at 570 nm on a multimode microplate reader (BioTek, Bedfordshire, UK).

### Wound healing assay

The transfected cells were plated in 12-well plates and grown to confluence. The cell monolayer was wounded using a 200-μl pipette tip. The wounds were imaged at 0, 24 and 48 h after wounding with a phase-contrast microscope (Olympus, Tokyo, Japan).

### Statistical analysis

GraphPad Prism version 8.0 was used to analyze all the experimental data. Differences between experimental groups were evaluated by a two-tailed Student's *t* test or one-way ANOVA. *P* values <0.05 were considered statistically significant.

## RESULTS

### Establishment of a novel engineered *T. Thermophila* ribozyme-based RNA circularization system

The desired RNA sequence to be circularized was inserted downstream of the optimized ribozyme and a complementary antisense region was added to the pCDH backbone upstream of the ribozyme to facilitate the folding and stabilization of the ribozyme. The catalytic domain in the 5' portion of the ribozyme was fused with an adjustable 13-nt-long IGS. The first 9 nt of the 3' terminus of the IGS sequence was complemented by pairing with the 3' terminus of the circRNA and a U residue was inserted in the 3' terminus of the circRNA. In contrast, the last 6 nt of the 5' terminus of the IGS was designed for incomplete complementary pairing with the 5' terminus of the target circRNA sequence to form helix P10. The secondary structure of RNA precursor was predicted by RNAFold (Supplementary Figure S1A).

**Figure 1.**
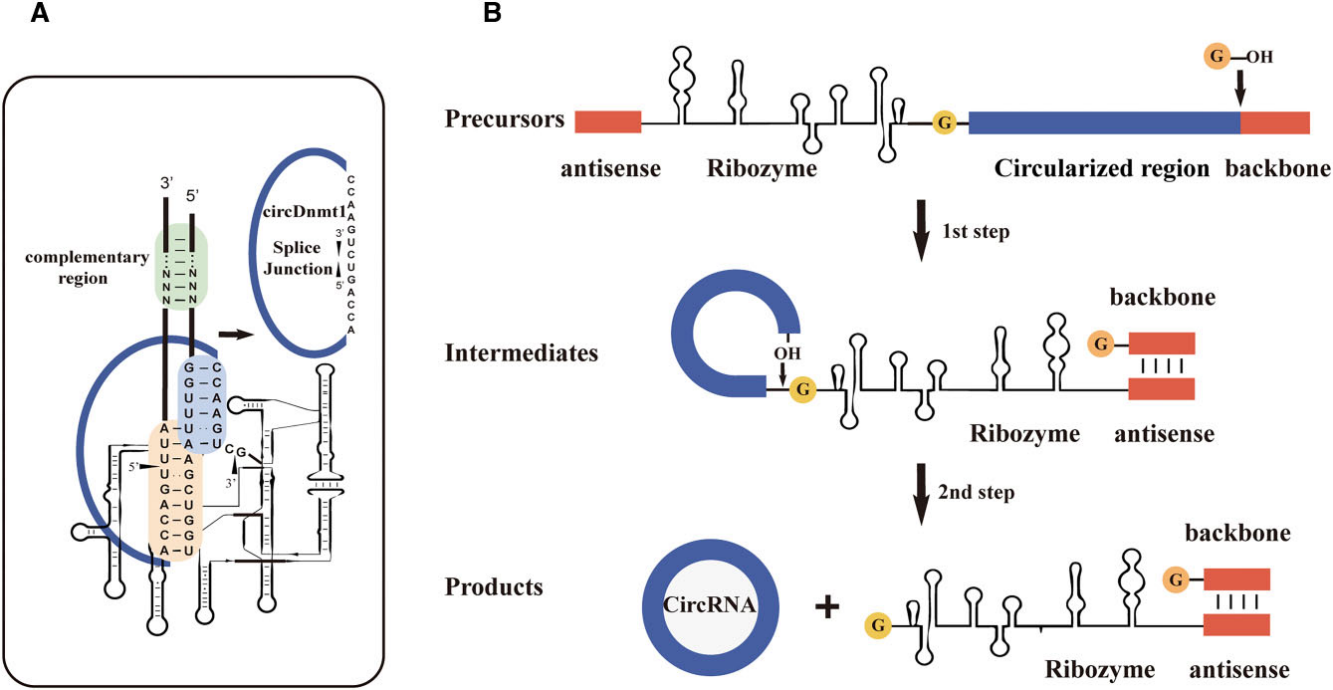
Construction of the *Tetrahymena* ribozyme-based circular expression construct. (**A**) Secondary structure of RNA transcribed from the *Tetrahymena* ribozyme-based circular construct for generation of circDNMT1. The 5' splice site, 3' splice site (black arrows), catalytic core of the *Tetrahymena* ribozyme showing the P1 (orange region), P10 (blue region), and complementary region (green region) are indicated. The complementary region is designed for ribozyme folding. RNA is cleaved at the 5' and 3' splice sites, and they are joined by sequential transesterification reactions, producing circDNMT1. (**B**) Schematic diagram showing the construct design and splicing process. The ribozyme-based circular construct consists of three parts: the antisense region complementary to the backbone (45 bp), the post-optimized sequence of the *Tetrahymena* ribozyme, and the desired RNA to be circularized. The 3' hydroxyl group of a guanosine nucleotide undergoes a transesterification reaction at the 5' splice site during splicing. The 3' terminal backbone sequence is removed, and the free hydroxyl group of the intermediate engages in a second transesterification reaction at the 3' splice site, leading to circularization of the intervening region and excision of the 5' antisense region and ribozyme.

**Figure 2.**
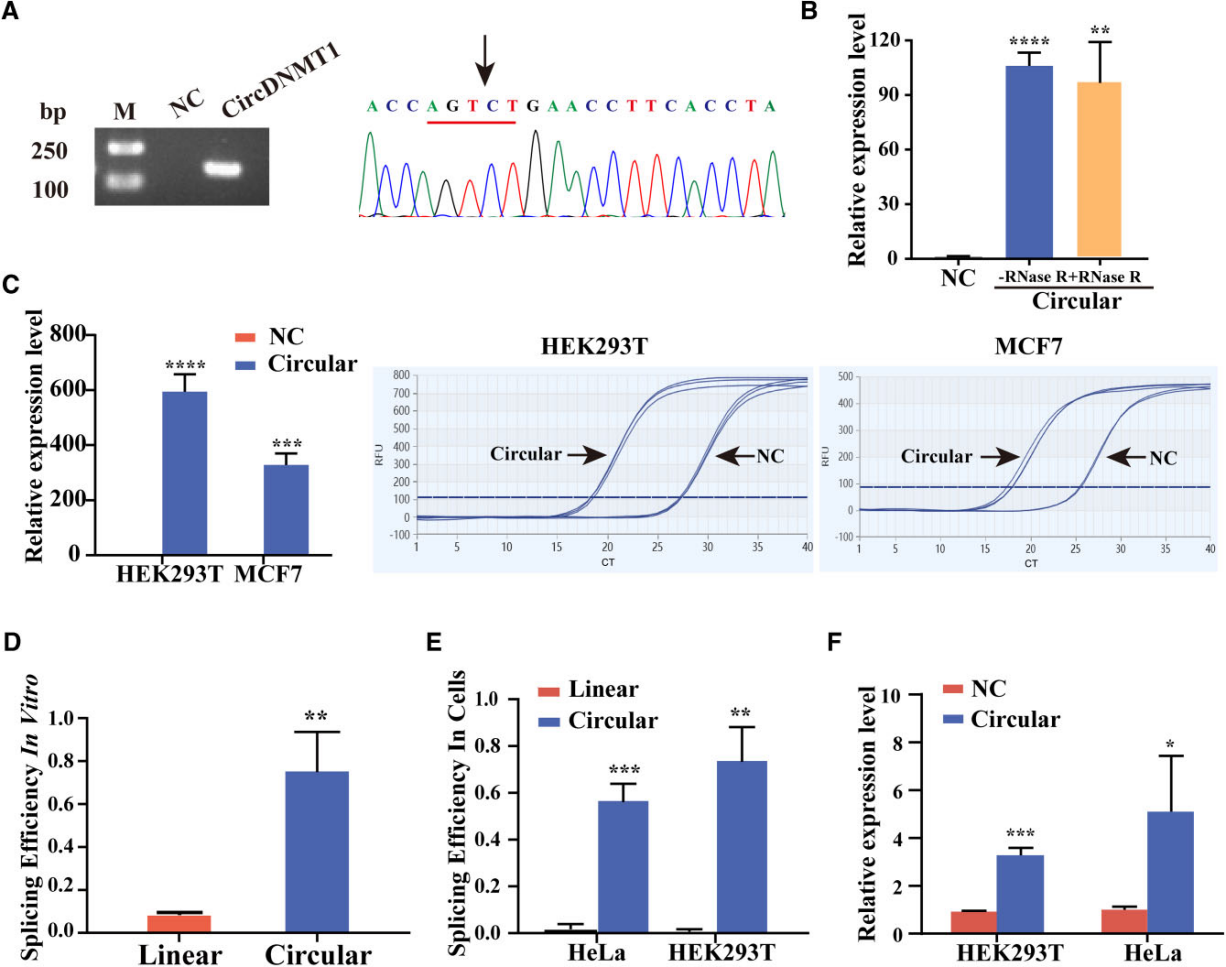
Efficiency evaluation of the circDNMT1 overexpression plasmid. (**A**) The overexpression of circDNMT1 was validated by RT-PCR followed by Sanger sequencing. The black arrow represents the ‘head-to-tail’ splicing site of circDNMT1. (**B**) The relative expression of overexpressed circDNMT1 treated with or without RNase R was calculated relative to the negative control group. (**C**) The expression level of circDNMT1 in HEK293T and MCF7 cells transfected with linear or circular expression constructs based on the *Tetrahymena* ribozyme. The amplification curves of qPCR are indicated. (**D**) The product with the T7 promoter was amplified from the circular or linear expression construct by RT-PCR using specific primers. The PCR products were transcribed *in vitro* using T7 RNA polymerase according to the manufacturer's instructions. The splicing efficiency of circDNMT1 with the linear or circular expression construct was calculated. The linear expression construct does not contain the ribozyme structure. The value was calculated by dividing the circular *DNMT1* expression level by the expression level of all *DNMT1* products (circular *DNMT1* ÷ total *DNMT1*). (**E**) After transfection of equal amounts of circular and linear expression constructs into HeLa and HEK293T cells, the splicing efficiency was analyzed by the same method. (**F**) The relative expression of circDNMT1 in HEK293T and HeLa cells using the flanking ICS-mediated circularization method. The values of all data shown are the means of three biological replicates. Error bars represent the standard deviations. Statistical significance was assessed using Student's *t* test (*P < 0.05, **P < 0.01, ***P < 0.001).

CircDNMT1 (hsa_circ_0049224) was chosen as a target circRNA to evaluate the established *T. thermophila* ribozyme-based RNA circularization system (Figure 1A). As a result, a free guanosine was covalently attached to the 3' terminus of *DNMT1* to produce circularized *DNMT1* (circDNMT1) (Figure 1B). The circDNMT1 overexpression plasmid and negative control plasmid were transfected into HeLa cells. Sanger sequencing revealed that the target RNA was circularized at the ‘U’ residue in the circDNMT1 natural splice junction (Figure 2A). qRT-PCR showed that the expression level of circDNMT1 was approximately 100 times higher than that of the negative control group in both the RNase R-treated group and the nontreated group (Figure 2B), and the circularization system achieved a 300–600-fold overexpression of circDNMT1 compared to the negative control group in HEK293T and MCF-7 cells (Figure 2C). Furthermore, the splicing efficiency of the RNA circularization method based on the *T. thermophila* ribozyme was approximately 80% *in vitro* (Figure 2D). Similar to the results of *in vitro*, *T. thermophila* ribozyme-based RNA circularization system realized a high splicing efficiency of close to 80% in HEK293T cells (Figure 2E). CircDNMT1 expression levels using the flanking ICS-mediated circularization method only increased 4–5-fold compared to those of the negative control group (Figure 2F).

### *T. Thermophila* ribozyme-based RNA circularization system enabled spontaneous circularization in mammalian cells

**Figure 3.**
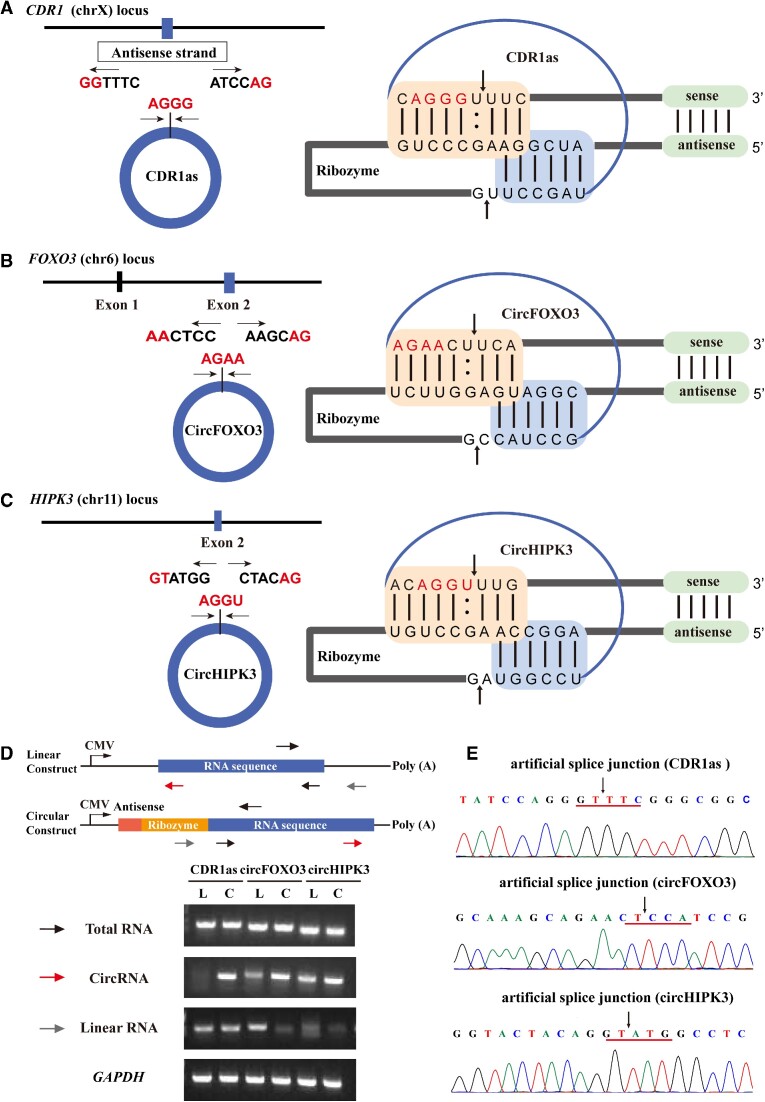
*T. thermophila* ribozyme-based RNA circularization system enabled spontaneous circularization in mammalian cells. (**A**–**C**) Schematic diagrams showing the genomic loci of three circRNAs in their host gene. Red fonts represent the natural splice junction of CDR1as, circFOXO3 and circHIPK3 (left). Schematic diagrams showing the base pairing between the circRNAs (CDR1as, circFOXO3 *and* circHIPK3) and the *Tetrahymena* ribozyme. The RNA sequences of the target circRNAs and ribozyme are shown, and helices P1 (orange) and P10 (blue) and the 45-nt-long complementary region (green) are indicated. (:) Shows wobble base pairs in the paired regions; arrows show the 5' and 3' splice sites (right). (**D**) Schematic diagram of primer design. Two versions of the constructs, with ribozyme or without ribozyme, were generated. The transcription of the constructs is driven by a CMV promoter and terminated by SV40 polyadenylation signal. Total RNA was purified 24 h after transfection into HEK293T cells and amplified by RT-PCR using various primer sets. The shared primers of the three primer pairs are indicated by the black arrows above. The black arrows, red arrows, and gray arrows below represent another pair of primers used for detecting total RNA, circRNA, and linear RNA, respectively (upper panel). Examination of total RNA, circRNA, and linear RNA of three circRNAs (CDR1as, circFOXO3, circHIPK3) in HEK293T cells transfected with linear expression constructs (L) and circular expression constructs (C), respectively (bottom panel). (E) The products were confirmed by Sanger sequencing. Arrows show the artificial splice sites of each circRNA.

CDR1as (hsa_circ_0001946), circFOXO3 (hsa_circ_0006404), and circHIPK3 (hsa_circ_0000284) were used to determine whether the *T. thermophila* ribozyme-based RNA circularization system was suitable for general circRNA overexpression. To avoid adding an extra uridine residue (‘U’) at the 5' splice site, we adjusted the IGS sequence to correctly hybridize the 5' and 3' terminal sequences of the rearranged circRNA sequence to form the P1 and P10 helices (Figure 3A–C, right). This adjustment causes circRNA to be spliced at the artificial site rather than the natural splicing site of circRNA (Figure 3A–C, left) but perfectly mimicked the native sequence of the circRNA.

**Figure 4.**
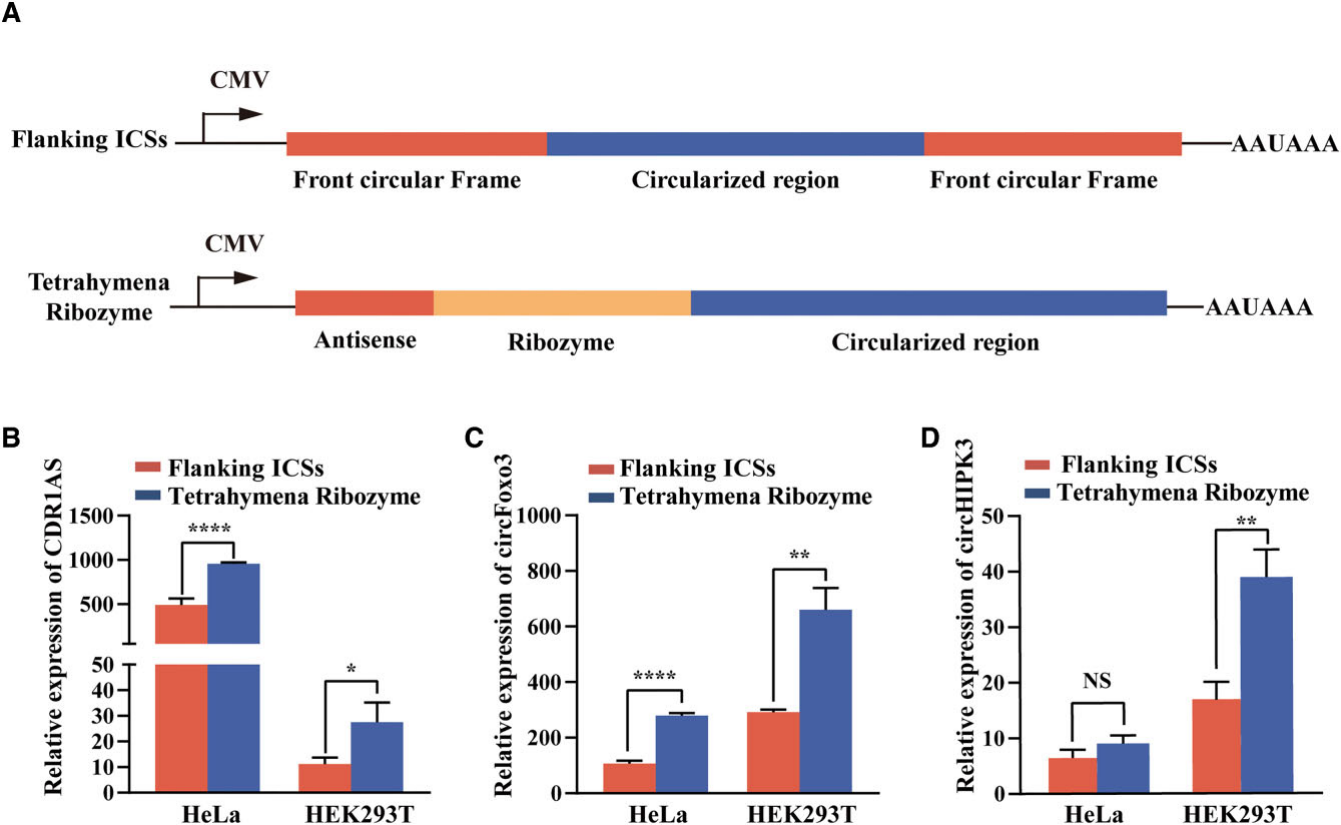
Evaluation of the overexpression effects of CDR1as, circFOXO3, and circHIPK3 using *Tetrahymena* ribozyme and flanking ICS method. (**A**) Schematic diagram of two plasmids of overexpressing circRNAs. For the overexpression plasmid based on flanking ICSs, the sequence for circRNA circularization was cloned in between the upstream and downstream complementary sequences. The flanking ICS method can promote the RNA circularization by back splicing (upper panel). For the overexpression plasmid based on the *Tetrahymena* ribozyme, the indicated RNA sequence was inserted downstream of the optimized ribozyme. The circRNA was generated by self-splicing of the ribozyme (lower panel). (**B**–**D**) The relative expression levels of CDR1as, circFOXO3, and circHIPK3 in HeLa and HEK293T cells transfected with two types of constructs as analyzed by qRT-PCR. The red bars represent flanking ICS method, and the blue bars represent *Tetrahymena* ribozyme-based circularization system. The results were normalized to the housekeeping gene *GAPDH* mRNA, and each value shows the fold change relative to the negative control group. The values shown are the means of three biological replicates. Error bars represent the standard deviations. Statistical significance was assessed using Student's *t* test (*P < 0.05, **P < 0.01, ***P < 0.001).

Divergent primers were designed for detecting circRNA. Convergent primers were designed for detecting total RNA including the forms of precursor RNA and circRNA. Linear primers were designed for detecting linear RNA that represented the RNA form that is not circularized in the precursor RNA (Figure 3D, upper panel). Negative control with the empty plasmid, linear expression constructs and ribozyme-based circular expression constructs were transfected into HEK293T cells. The endogenous or overexpressed circRNAs in HEK293T cells were detected in all groups by RT-PCR (Supplementary Figure S1B). The results showed that our ribozyme-based circular constructs strongly enhanced the production of circRNA compared with endogenous levels. Furthermore, compared with the linear expression constructs, the circular expression constructs achieved higher circRNA expression in the case of plasmid transfection of the same quality (Figure 3D, lower panel). The sequence correctness of the artificial site of end-joining was verified by Sanger sequencing (Figure 3E). The overexpression plasmids based on ribozyme (Figure 4A, lower panel) or flanking ICSs (Figure 4A, upper panel) were constructed and the relative expression levels of circRNAs overexpressed by different circularization methods were evaluated in HeLa and HEK293T cells (Figure 4B–D). The results showed that the *T. thermophila* ribozyme-based RNA circularization system had overwhelming advantages over the commonly used flanking ICS method.

### CircRNAs synthesized by *T. thermophila* ribozyme-based RNA circularization system performed biological functions consistent with those of natural noncoding circRNAs

**Figure 5.**
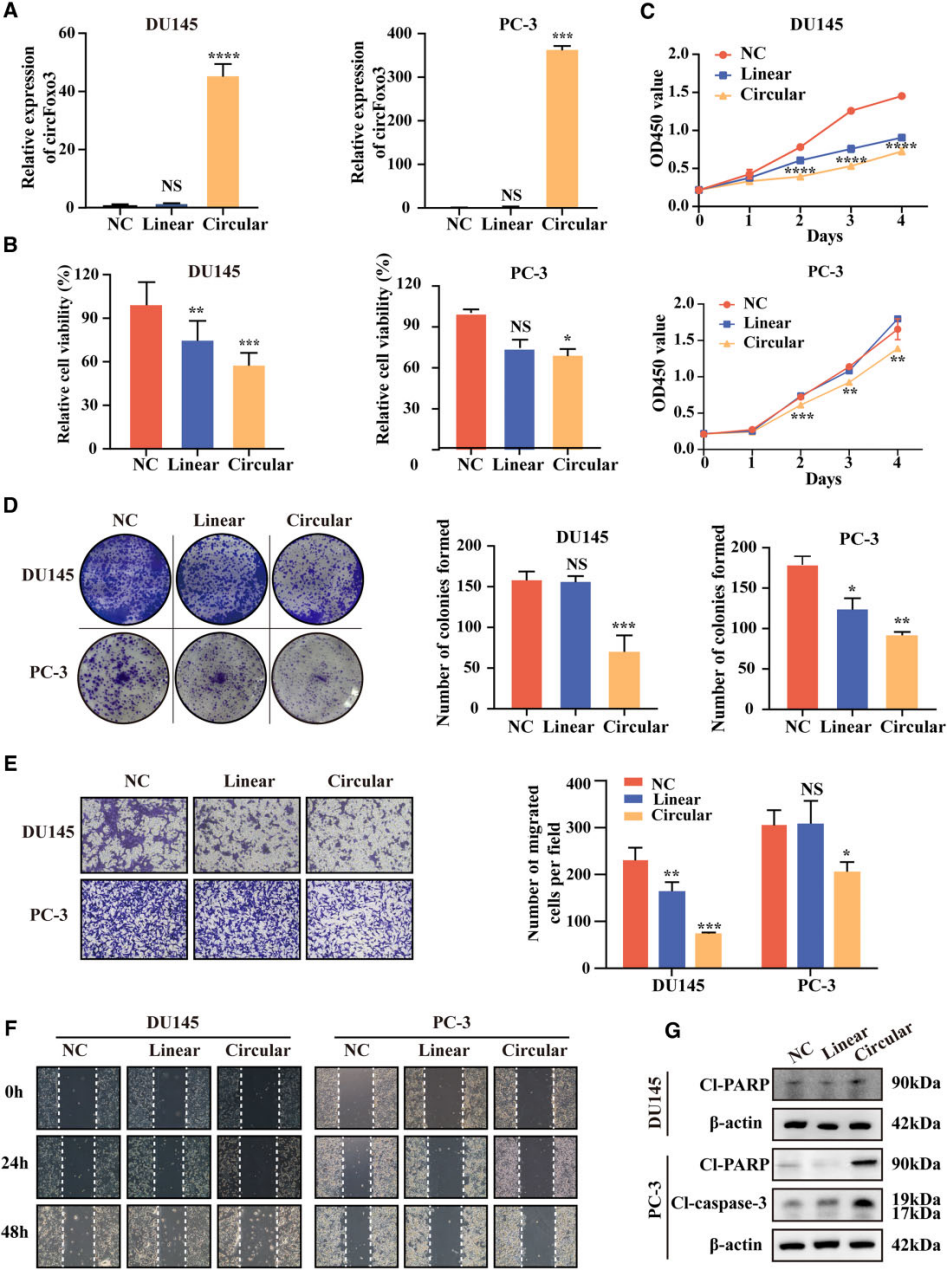
Overexpression of circFOXO3 using our strategy regulated cell proliferation, migration, and apoptosis of prostate cancer cells. (**A**) The relative expression levels of circFOXO3 in DU145 and PC3 cells transfected with negative control (pCDH vector), linear and circular expression constructs were analyzed by qRT-PCR. Statistical significance was assessed using one-way ANOVA (*P < 0.05, **P < 0.01, ***P < 0.001). (**B**) Cell viability was measured using Cell Counting Kit-8 (CCK-8) assays. Statistical significance was assessed using one-way ANOVA (*P < 0.05, **P < 0.01, ***P < 0.001). (**C**) CCK-8 assays were performed to examine the proliferation ability of DU145 and PC3 cells. Statistical significance was assessed using Student's *t* test (*P < 0.05, **P < 0.01, ***P < 0.001). (**D**) The transfected DU145 and PC3 cells were subjected to clone formation assays for 14 days. Left, typical images of clone formation. Right, quantification of clone formation revealing that circFOXO3 overexpression suppressed cell proliferation. The number of cell clones was normalized to the number of cells counted at the endpoint. Statistical significance was assessed using one-way ANOVA (*P < 0.05, **P < 0.01, ***P < 0.001). (**E**) The transfected DU145 and PC3 cells were subjected to transwell migration assays for 24 h. Left, typical images showing typical cell migration after 24 h. Right, quantification of transwell migration assays showing that the expression of circFOXO3 inhibited cell migration. The number of migrating cells was normalized to the number of cells counted at the endpoint. Statistical significance was assessed using one-way ANOVA (*P < 0.05, **P < 0.01, ***P < 0.001). (**F**) Wound healing assay showing that overexpression of circFOXO3 inhibited the migration of DU145 and PC3 cells. (**G**) Western blots showing that the overexpression of circFOXO3 increased the expression of apoptosis-related proteins induced by cisplatin in DU145 and PC3 cells. All data are presented as the means of three biological replicates. Error bars represent the standard deviations.

Both DU145 and PC-3 cells were transfected with circFOXO3 linear and circular expression constructs separately to observe the intracellular function of the *T. thermophila* ribozyme-based circularization system (Figure 5A). As expected, the high expression of circFOXO3 suppressed the viability (Figure 5B), proliferative activity (Figure 5C), and clone formation ability (Figure 5D) of DU145 and PC-3 cells compared to the negative control vector and linear expression construct groups. The transwell migration and wound healing assays also showed that the overexpression of circFOXO3 suppressed the migration activities of DU145 and PC-3 cell lines (Figure 5E, F). Moreover, the overexpression of circFOXO3 promoted cisplatin-induced apoptosis (Figure 5G).

CDR1as, which is associated with the occurrence of various cancers, has more than 60 binding sites for miR-7. It has been suggested that CDR1as may function as a miR-7 sponge (27). We investigated whether CDR1as maintains regulation of miR-7 function when overexpressed using the *T. thermophila* ribozyme-based *c*ircularization system in cells expressing miR-7 (28). The CCK8 assay, transwell migration assay, and colony formation assay showed that CDR1as overexpression reduced miR-7 tumor suppressive function (Supplementary Figure S2), which was in line with similar observations by Weng *et al.* (28), highlighting that the system successfully mimics native CDR1as. However, it should be noted that it has been shown that CDR1as is not expressed in the cancer cells in colon tumors from patients (29).

### *T. Thermophila* ribozyme-based RNA circularization system promoted RNA circularization *in vitro* and performed the same protein expression function as mRNA

**Figure 6.**
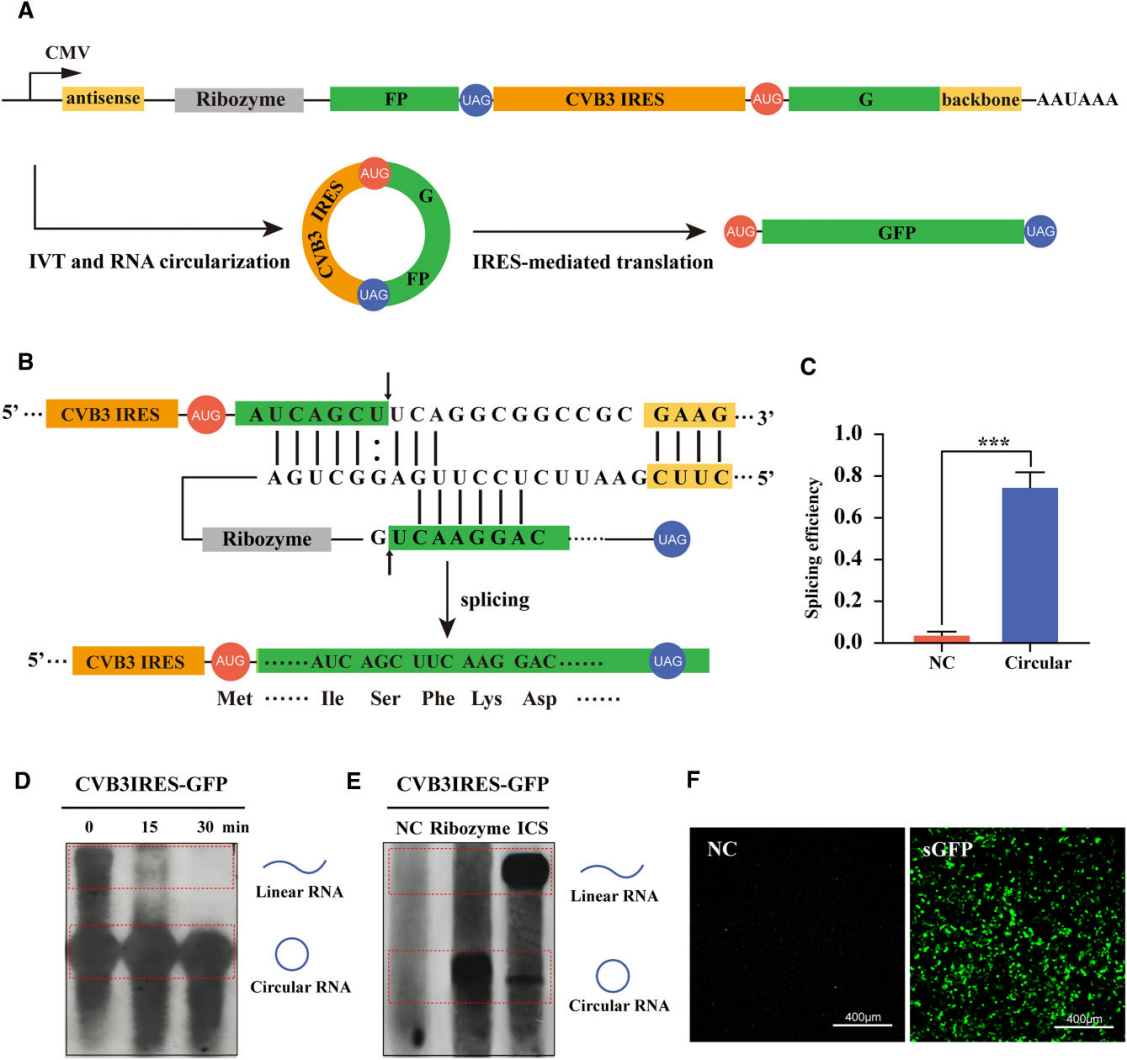
Evaluation of the circularization efficiency and translation of GFP-coding circRNA using *T. thermophila* ribozyme-based circularization system. (**A**) Schematic diagram showing the vector with the split GFP sequence inserted in reverse order. The transcription of the vector was driven by a CMV promoter and terminated by an SV40 polyadenylation signal. (**B**) Schematic diagram showing the base pairing between the precursor RNA and ribozyme. Helices P1 and P10 and the complementary region are indicated. (:) shows wobble base pairs, and arrows show the 5' and 3' splice sites. The predicted translation product mediated by CVB3 IRES is shown. The ligated sequence was verified by Sanger sequencing (an arrow indicates the splicing site). (**C**) Two pairs of primers were used to detect total or circular RNAs by qRT-PCR. The splicing efficiency of the circular RNA encoding GFP was calculated. The values shown are the mean of three biological replicates. Error bars represent the standard deviations. Statistical significance was assessed using Student's *t* test (*P < 0.05, **P < 0.01, ***P < 0.001). (**D**) Time course splicing of Tetrahymena ribozyme on CVB3IRES-GFP performed by northern blot. The circularization time points were set at 0 min, 15 min and 30 min. The hybridization probe with GFP sequence labelled with digoxin was synthesized to detect precursor linear RNAs and circRNAs. (**E**) The circularization efficiency of CVB3IRES-GFP was compared between two circularization systems based on Tetrahymena ribozyme and the flanking ICS method by northern blot. (**F**) GFP fluorescence in HEK293T cells 48 h after transfection with circRNA encoding GFP synthesized by our system (scale bar: 400 μm).

To verify whether circRNA could be used as a tool for protein translation based on the *T. thermophila* ribozyme-based circularization system, a gene reporter containing two GFP fragments in a reversed order was engineered. CVB3 IRES from previous research was engineered upstream of the start codon of GFP to drive cap-independent protein synthesis (Figure 6A) (30). A schematic representation of the ribozyme and split GFP complex is shown in Figure 6B. The circularization efficiency of the circular sequence encoding GFP was significantly higher than that in the negative control group (Figure 6C). We performed the time-course experiment to reveal the *in vitro* splicing process with northern blot analysis, as shown in Figure 6D. Under the optimized splicing conditions as reported, we compared the multiple circularization conditions (55°C for 0 min, 15 min and 30 min, respectively). The results showed that most transcribed RNAs had been circularized during *in vitro* transcription, suggesting that the transcription initiated splicing and that a further 15-min incubation was sufficient after the transcription, which is consistent with the reaction conditions in most studies (26,31). Similarly, we transfected the CVB3IRES-GFP overexpression plasmid into HEK293T cells and used NB to compare these two methods. The ratio of circRNA to precursor linear RNA of the *T. thermophila* ribozyme was much higher than that of the classic flanking ICS method, suggesting higher splicing efficiency of the ribozyme system (Figure 6E). GFP fluorescence was observed from the transfection of circRNA formed, which proved that the CVB3 IRES and split GFP sequences could be joined together by backsplicing (Figure 6F). Moreover, 5-methylcytosine and 2'-O-methyladenosine were introduced into synthetic circRNAs, demonstrating that nucleoside modifications would not disrupt the production of circRNA (Supplementary Figure S3).

## DISCUSSION

Efficient and precise preparation of circRNAs is important for the functional study of circular mRNA pharmaceutics in the future. Previous strategies for circRNA synthesis did not meet the requirements due to their inefficiency and the introduction of additional nucleotides. Therefore, it is necessary to further simplify the procedure and improve production efficiency to achieve industrial-scale production. In our current study, the *T. thermophila* ribozyme-based circularization system achieved a circularization efficiency of 80% *in vitro* and in mammalian cells, and the circRNA produced by this system was completely consistent with the designed sequence without introducing any additional nucleotides. Due to the complexity of the intracellular environment in mammalian cells, the splicing efficiency of ribozyme varies with gene sequence and cell type. In recent years, the ribozyme derived from the *T. thermophila* group I intron has been widely applied in gene delivery, mRNA repair, and mRNA detection (32,33). Lieshout et al. previously used the *Tetrahymena* ribozyme to synthesize long circular RNA in *E. coli* based on the PIE strategy, and this circular transcript could be translated into an active β-glucosidase (34). However, due to suboptimal assembly of the ribozyme catalytic domain in the PIE strategy, intron truncation may lead to a lower splicing efficiency. In our present study, the new system inserts the target RNA sequence downstream of the optimized ribozyme, which is simple to perform and retains the complete ribozyme sequence and high splicing efficiency. Second, our system simulated three endogenous circRNAs and verified their biological functions in mammalian cells. The results showed that this system could spontaneously catalyze the synthesis of endogenous circRNA in mammalian cells and maintain its original biological function.

Because of its covalently closed circular structure, circular mRNA is more resistant to endonuclease degradation and more stable than linear RNA (35), thus producing higher levels of and longer-lasting proteins (15). Therefore, engineering circRNA for translation as mRNA may be an effective protein expression tool that can be applied to clinical disease treatment. The products transcribed by our circularization system can be rapidly self-catalyzed *in vitro* to produce a large number of precise circRNAs as protein overexpression vectors through simplified reaction and purification processes. We used the GFP reporter system to further verify the possibility of our RNA circularization system acting as an mRNA for translation. The coding region of GFP was divided into two parts: the first part containing the initiation codon was preceded by an IRES sequence, which could act as an mRNA cap structure in mammalian cells. The second part of GFP was located in front of the IRES and behind the self-splicing ribozyme. Without the introduction of additional nucleotides, the coding sequence of GFP will not frameshift and green fluorescence will be expressed under the mediation of IRES. For any circRNA synthesized using the ribozyme described here, it is necessary to prevent the end-joining sequence from becoming a stop codon to ensure the translation of the full-length circRNA. Recently, it has been reported that exogenous circRNA was prepared by the PIE strategy *in vitro* to produce neutralizing antibodies against SARS-CoV-2 (31). In contrast, the circRNA generated by our ribozyme circularization system does not contain redundant sequences, which may reduce unexpected deleterious effects *in vivo*.

Recent research has indicated that the nucleotide modification of circRNAs attenuates the innate immune response and may enhance the future applicability of circRNAs in treatment (36–38). Therefore, nucleoside modification should be incorporated into the production strategy of circRNA production. In our current study, we also demonstrated that nucleoside modifications did not affect the circularization efficiency of circRNA production, which may be used to generate circRNA with the potential to become an effective and safe platform for RNA therapy (Supplementary Figure S3). Chen *et al.* developed a systematic approach to rapidly assemble and test elements engineering circular RNA for enhanced protein production. CircRNA protein production was increased several hundred-fold by optimizing vector topology, 5' and 3' untranslated regions, IRES, and synthetic aptamers recruiting translation initiation machinery (39), which provides new insight for us to increase the production of circRNA protein in the future. We believe that our strategy will facilitate the application of circRNAs as mRNAs for disease treatment and prevention.

In conclusion, the circRNA produced by our *T. thermophila* ribozyme-based circularization system can mimic the natural noncoding circRNA and participate in various intracellular functions, and may also be used in the preparation of circular mRNAs to express therapeutic proteins. Because of its unique characteristics, circRNA may become a safe and effective choice for therapeutic use as well as vaccination against viral infection, including new SARS-CoV-2 variants. We believe this novel RNA circularization system will soon have broad industrial, scientific, and clinical applications.

## Supplementary Material

gkad554_Supplemental_Files

## Data Availability

The data that support the findings of this study are available from the corresponding author upon reasonable request.
