# Supplementary material for: A precise and efficient circular RNA synthesis system based on a ribozyme derived from Tetrahymena thermophila
Source: Nucleic Acids Res. 2023 Jun 28;51(14):e78. doi: 10.1093/nar/gkad554 (PMC10415121; doi:10.1093/nar/gkad554)
Supplement: gkad554_Supplemental_Files [file gkad554_supplemental_files.zip › supplementary figures.pdf]

Supplementary Figure

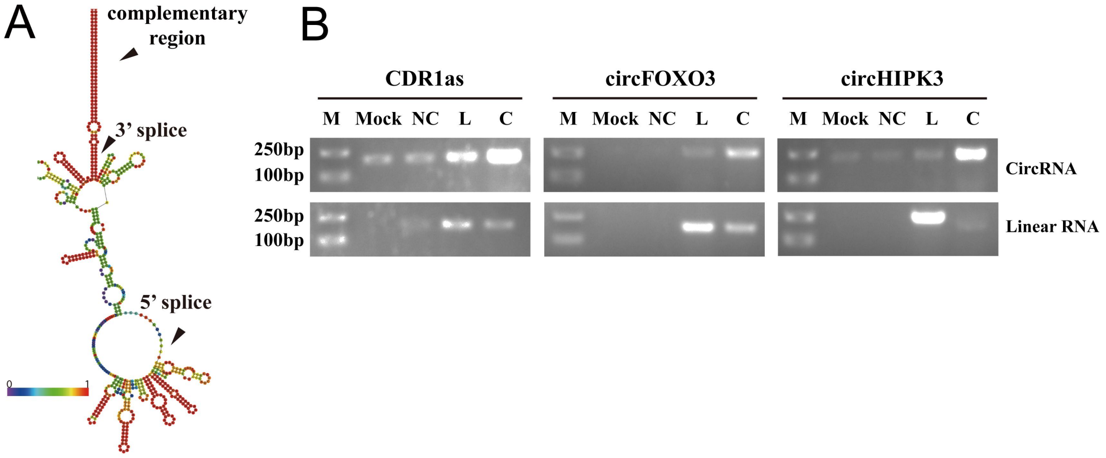

Supplementary Figure 1. Validation of circularization mediated by Tetrahymena ribozyme. (A) RNAFold (http://rna.tbi.univie.ac.at/cgi-bin/RNAWebSuite/RNAfold.cgi) was used to predict the precursor RNA secondary structure. The colors indicate the probability of base pairing, with red suggesting a higher probability. (B) Examination of circRNAs and linear RNAs of three circRNAs (CDR1as, circFOXO3, circHIPK3) in untransfected HEK293T cells (Mock) and cells transfected with negative control vector (NC), linear expression construct (L) and ribozyme-based circular expression construct (C), respectively.

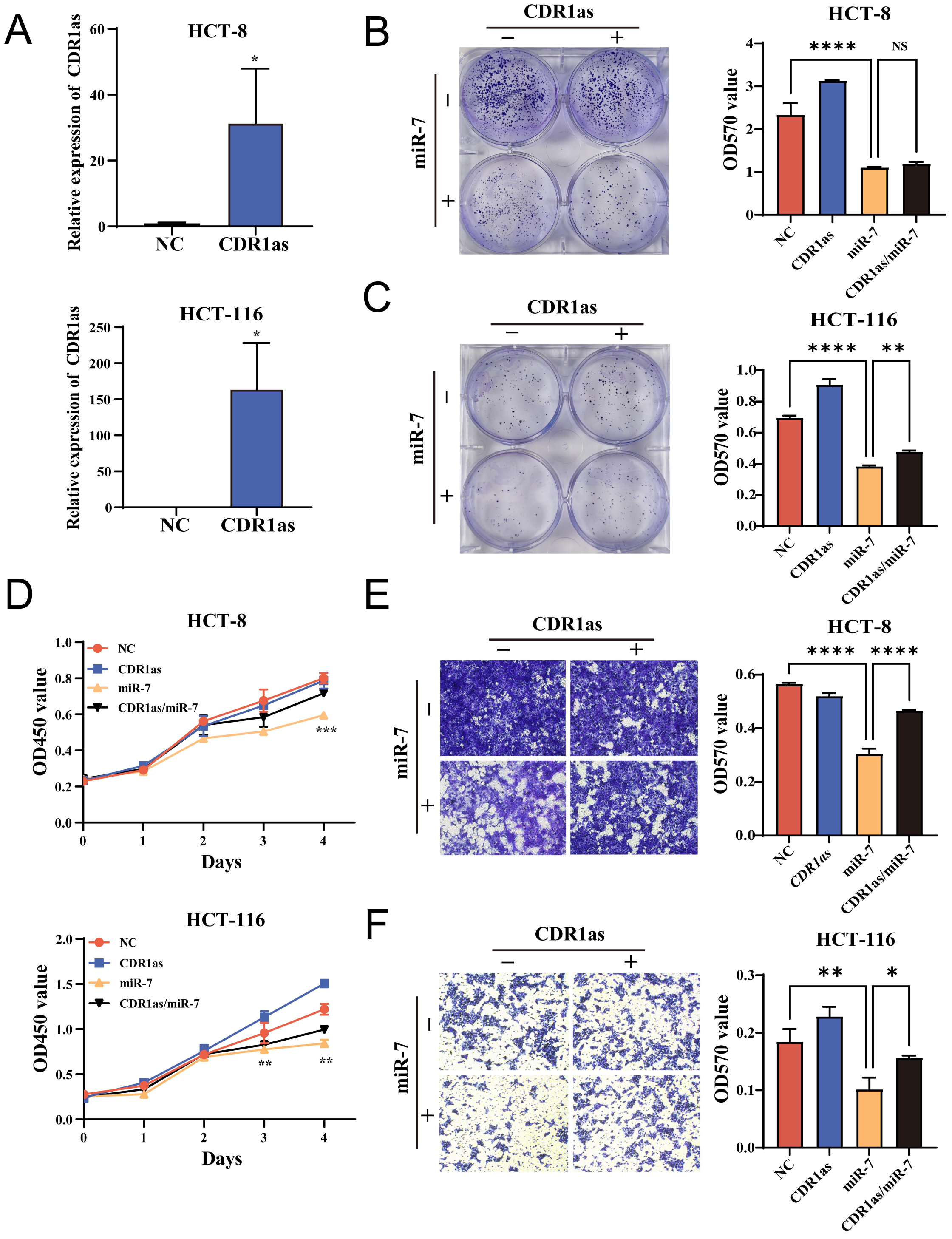

Supplementary Figure 2. CDR1as inhibited the tumor suppressive effects of miR-7 in colorectal cancer cells. (A) The effect of overexpression of CDR1as by tetrahymena ribozyme was verified by qPCR. Statistical significance was assessed using Student's *t* test (**P* < 0.05, ***P* < 0.01, ****P* < 0.001). (B, C) Colony formation assay showed CDR1as overexpression partly rescued inhibition of cells proliferation ability, which was induced by

miR-7. Statistical significance was assessed using one-way ANOVA (*P < 0.05, **P < 0.01, ***P < 0.001). (D) CCK8 assay was performed in HCT-8 and HCT-116 cells with overexpression of miR-7 alone, CDR1as alone or both. Statistical significance was assessed using Student's *t* test (**P* < 0.05, ***P* < 0.01, ****P* < 0.001). (E, F) Transwell migration assay showed miR-7 overexpression inhibited migration ability of HCT-8 and HCT-116 cells, which was partly neutralized by CDR1as overexpression. Statistical significance was assessed using one-way ANOVA (**P* <0.05, ***P* < 0.01, ****P* < 0.001). All data presented as the means of three biological replicates. Error bars represent the standard deviations.

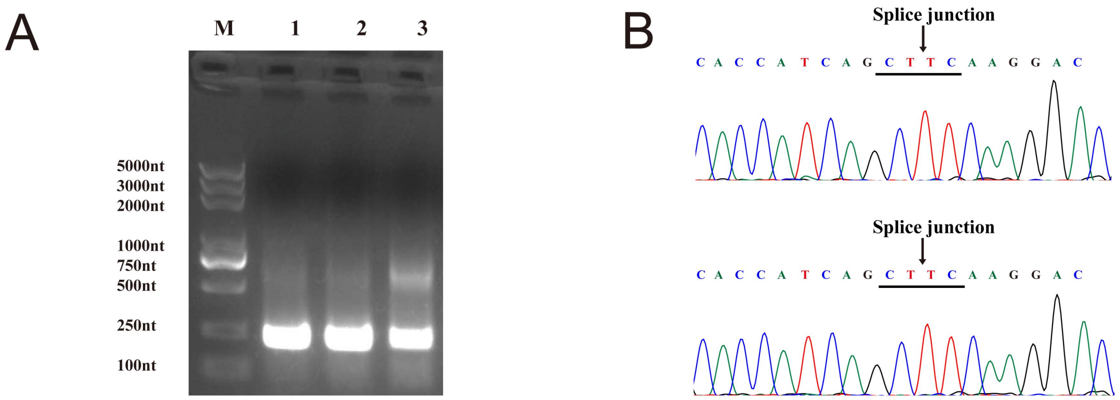

Supplementary Figure 3. Chemical modification of nucleotides does not affect circularization of the *Tetrahymena* ribozyme system. (A) The splice junction site of the circRNA with nucleoside modification was characterized by Sanger sequencing following amplification by RT-PCR. M, marker. Lane 1, the generated circRNA without nucleoside modification. Lane 2, the generated circRNA with 5-methylcytosine (m5C) modification. Lane 3, the generated circRNA with 2'-O-methyladenosine modification. (B) The ligated sequence was verified by Sanger sequencing (an arrow indicates the splicing site).
